# Development of a multimodal vision transformer model for predicting traumatic versus degenerative rotator cuff tears on magnetic resonance imaging: A single‐centre retrospective study

**DOI:** 10.1002/ksa.70000

**Published:** 2025-08-13

**Authors:** Felix C. Oettl, Ali B. Malayeri, Pascal R. Furrer, Karl Wieser, Philipp Fürnstahl, Samy Bouaicha

**Affiliations:** ^1^ Department of Orthopedic Surgery Balgrist University Hospital, University of Zürich Zurich Switzerland; ^2^ Research in Orthopedic Computer Science Group Balgrist University Hospital, University of Zurich Zurich Switzerland

**Keywords:** artificial intelligence, magnetic resonance imaging, rotator cuff tear, shoulder surgery, vision transformer

## Abstract

**Purpose:**

The differentiation between traumatic and degenerative rotator cuff tears (RCTs remains a diagnostic challenge with significant implications for treatment planning. While magnetic resonance imaging (MRI) is standard practice, traditional radiological interpretation has shown limited reliability in distinguishing these etiologies. This study evaluates the potential of artificial intelligence (AI) models, specifically a multimodal vision transformer (ViT), to differentiate between traumatic and degenerative RCT.

**Methods:**

In this retrospective, single‐centre study, 99 shoulder MRIs were analysed from patients who underwent surgery at a specialised university shoulder unit between 2016 and 2019. The cohort was divided into training (*n* = 79) and validation (*n* = 20) sets. The traumatic group required a documented relevant trauma (excluding simple lifting injuries), previously asymptomatic shoulder and MRI within 3 months posttrauma. The degenerative group was of similar age and injured tendon, with patients presenting with at least 1 year of constant shoulder pain prior to imaging and no trauma history. The ViT was subsequently combined with demographic data to finalise in a multimodal ViT. Saliency maps are utilised as an explainability tool.

**Results:**

The multimodal ViT model achieved an accuracy of 0.75 ± 0.08 with a recall of 0.8 ± 0.08, specificity of 0.71 ± 0.11 and a F1 score of 0.76 ± 0.1. The model maintained consistent performance across different patient subsets, demonstrating robust generalisation. Saliency maps do not show a consistent focus on the rotator cuff.

**Conclusion:**

AI shows potential in supporting the challenging differentiation between traumatic and degenerative RCT on MRI. The achieved accuracy of 75% is particularly significant given the similar groups which presented a challenging diagnostic scenario. Saliency maps were utilised to ensure explainability, the given lack of consistent focus on rotator cuff tendons hints towards underappreciated aspects in the differentiation.

**Level of Evidence:**

Not applicable.

**Abbreviations**
AI: artificial intelligence
AUC‐ROC: area under the curve‐receiver operating characteristic
BMI: body mass index
CI: confidence interval
CNN: convolutional neural networks
ML: machine learning
MRI: magnetic resonance imaging
RCT: rotator cuff tears
ViT: vision transformer

## INTRODUCTION

Rotator cuff tears (RCTs) are frequently encountered in orthopaedic practice and represent a significant clinical and socioeconomic burden [18, 19, 22]. RCTs can lead to pain, disability, and challenges in determining the underlying aetiology—whether the tear is traumatic or degenerative—remains unresolved [4]. This distinction is crucial, as it influences treatment strategies, urgency and cost coverage by insurance providers [4, 8]. However, accurately classifying RCTs remains challenging due to the degenerative changes of the rotator cuff over time. Traditional diagnostic approaches rely on patient history, risk factors such as age, and imaging features like muscle atrophy, tendon kinking and oedema, as well as the newly established Cobra Sign [4, 6]. While magnetic resonance imaging (MRI) and magnetic resonance arthrograms offer valuable insights, distinguishing between traumatic and degenerative RCTs based on imaging alone remains difficult [6, 14, 16, 25].

Artificial intelligence (AI) and machine learning (ML) are revolutionising medical imaging, including musculoskeletal medicine [5, 10, 13]. These technologies have been successfully applied to orthopaedic diagnostics [1, 11, 17, 24]. AI‐driven models have the potential to enhance RCT classification by automating MRI interpretation, reducing interobserver variability and improving diagnostic precision, by leveraging deep learning algorithms, AI can extract complex imaging features [1, 11, 12, 17, 24].

This study is a pilot, aiming to develop and validate a ML model capable of automatically distinguishing between traumatic and degenerative RCTs based on MRI scans in combination with basic demographic data. By integrating deep learning with structured clinical data, it seeks to enhance diagnostic accuracy and take the first step to provide clinicians with a robust, data‐driven clinical decision support (CDS) tool. It was hypothesised that a multimodal AI model, integrating both MRI data and patient demographic features, would differentiate between traumatic and degenerative RCT with significantly greater accuracy than an AI model based on imaging alone. The development of such a tool is clinically relevant as it could provide a data‐driven CDS system to optimise treatment strategies and improve patient outcomes.

## METHODS AND MATERIALS

This retrospective analysis was conducted at a specialised university shoulder unit, analysing shoulder MRI arthrograms from patients treated between 2016 and 2019. The dataset comprised 99 MRI scans from patients with full‐thickness RCTs, divided into traumatic and degenerative groups with similar age and affected rotator cuff muscles. The study was conducted with institutional review board approval and informed patient consent.

The traumatic group included patients who experienced a relevant shoulder trauma and were previously asymptomatic. Minor injuries such as simple lifting traumas were specifically excluded. For inclusion, patients required MRI arthrography within 3 months of the traumatic event. The degenerative group consisted of age‐ and affected tendon‐matched patients reporting chronic shoulder pain for at least 1 year prior to MRI, with no history of trauma to the affected shoulder. Patients over 66 years of age, those with previous operations on the affected shoulder, and cases showing Goutallier Grade II or higher fatty infiltration in the ruptured muscle were excluded (Table 1).

**Table 1:** Demographics of the degenerative and traumatic patients.

	Degenerative	Traumatic	*p* value
Age (years)	61.2 ± 8.1	63.4 ± 5.7	n.s.
Height (cm)	171.0 ± 10.95	167.6 ± 9.69	n.s.
Weight (kg)	77.98 ± 17.80	78.52 ± 15.39	n.s.
BMI	26.49 ± 4.63	27.94 ± 5.03	n.s.
Smoker (%)	78	64.0	n.s.

*Note*: Data are presented as mean ± SD unless otherwise specified.

Abbreviation: BMI, body mass index.

For model development and validation, the dataset of 99 MRI scans was divided into a training set of 79 scans and a test set of 20 scans. To ensure standardisation across the dataset, each patient's MRI series was processed to include the 20 centre slices, either by padding cases with fewer slices or selecting a subset from those with more slices. Each slice was treated as an individual training sample, with the patient's overall classification label assigned to all slices. The vision transformer (ViT) and ResNet models were trained on these 2D slices independently, and in the multimodal model, each slice was paired with the corresponding patient's demographic data.

### Preprocessing

Images were extracted from DICOM files and underwent pixel intensity normalisation and were resized to a uniform resolution of 224 × 224 pixels. To enhance the robustness of the models and expand the effective size of the training dataset, extensive data augmentation was implemented [2]. The augmentation protocol included rotations of 40 degrees, width and height shifts of 20%, shear transformations of 20%, zoom adjustments of 20%, horizontal flipping and random contrast adjustments ranging from 0.8 to 1.2. During these transformations, nearest neighbour interpolation for pixel filling to maintain image integrity was employed [23].

Each MRI scan comprises 20 centre slices, with each slice treated as an independent input for training. The model predicts the label for each slice individually. During inference, a final patient‐level classification is determined using ensemble averaging across the 20 slice‐level predictions, ensuring a more robust decision‐making process [20].

### Training

**Figure 1:**
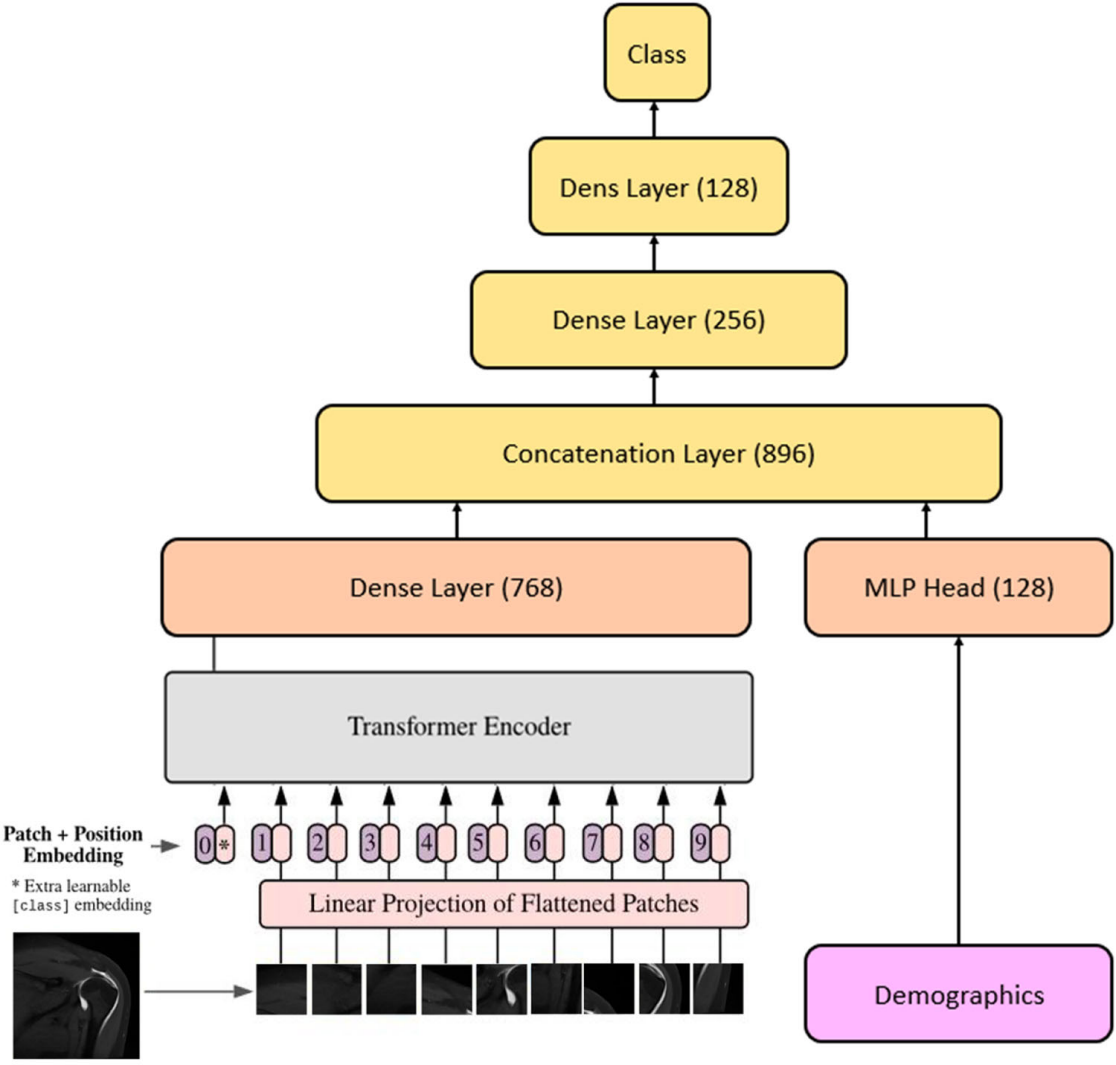
Architecture of the multimodal model combining a vision transformer (ViT) with demographic data. The model processes medical imaging using a ViT, where image patches undergo linear projection and positional embedding before being passed through a transformer encoder. The extracted image features of merged with demographic data via a concatenation layer, followed by fully connected layers leading to the final classification output.

Three distinct approaches for classification were explored. Initially, a ResNet80 model pretrained on ImageNet was implemented [9]. To accommodate the grayscale MRI images, a preprocessing layer to convert them to three‐channel format compatible with the ResNet80 architecture was implemented. This model was trained using the Adam optimiser with a learning rate of 1 × 10^−7^, employing a batch size of 16 over 200 epochs, using binary cross‐entropy as the loss function. The second approach utilised a ViT model, which was trained with slightly different parameters [3]. The ViT implementation used the Adam optimiser with a learning rate of 1 × 10^−5^, a larger batch size of 32, and ran for 60 epochs, also using binary cross‐entropy loss function. Finally, a multimodal approach that integrated demographic information with imaging features was developed. This model first processed MRI scans through the ViT architecture to generate a 678‐dimensional feature vector. Simultaneously, it processed 17 demographic features through a separate network of fully connected layers with ReLU activation. These two feature sets were then concatenated and refined through additional dense layers before final classification (Figure 1) [21].

All models were evaluated using 5‐fold cross‐validation, ensuring a consistent split of 79 training patients and 20 validation patients in each fold. Since each MRI scan contains 20 slices, predictions were first made at the slice level and then aggregated at the patient level using ensemble averaging. This approach enabled a robust evaluation of model performance across different patient subsets. Performance was assessed using standard metrics, including precision, recall, specificity, F1 score and accuracy.

To enhance interpretability and assess model decision‐making, saliency maps using a direct, gradient‐based visualisation approach (often referred to as ‘vanilla saliency maps’) were generated. This method computes the gradients of the predicted class score with respect to the input pixels, highlighting the most influential regions in the image. Specifically, a gradient tape to backpropagate the prediction error and then aggregate the absolute values of these gradients to form heatmaps was utilised. By superimposing these heatmaps onto the original images, one can visually discern which areas the model deems most relevant for classification.

This study was conducted with review board approval from the Kantonale Ethikkommision Zürich (Cantonal ethics committee of Zurich), approval number 2020‐02647, granted on 13 September 2024.

## RESULTS

**Figure 2:**
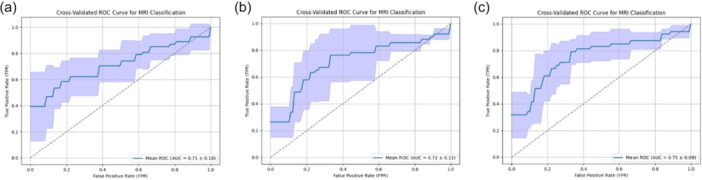
Receiver operating characteristic (ROC) curves for three models: (a) a ResNet‐based model, (b) a vision transformer (ViT) and (c) a multimodal model combining ViT with demographic data. The area under the curve (AUC) quantifies model performance.

The multimodal model incorporating MRI features from the ViT and patient demographics achieved the highest performance among the evaluated architectures. Specifically, this model attained an accuracy of 74%, outperforming ViT alone (71%) and ResNet50 (70%). Precision, recall, specificity and F1‐score followed similar trends, with the multimodal approach consistently yielding superior values (Figure 2, Table 2).

**Table 2:** Performance of machine learning models.

	ResNet80	ViT	Multimodal model
Precision	0.74 ± 0.06	**0.8 **±** 0.19**	0.73 ± 0.14
Recall	0.59 ± 0.12	0.64 ± 0.08	**0.75 **±** 0.14**
Specificity	**0.79 ± 0.1**	0.79 ± 0.2	0.72 ± 0.09
F1 score	0.65 ± 0.09	0.69 ± 0.11	**0.74 ± 0.12**
Accuracy	0.7 ± 0.08	0.71 ± 0.11	**0.74 ± 0.08**
AUC‐ROC	0.71 ± 0.1	0.72 ± 0.11	**0.75 ± 0.09**

*Note*: Performance metrics are displayed as point performance ± SD. The bold Values highlight the best model for the respective metric.

Abbreviations: AUC‐ROC, area under the curve‐receiver operating characteristic; ViT, vision transformer.

**Figure 3:**
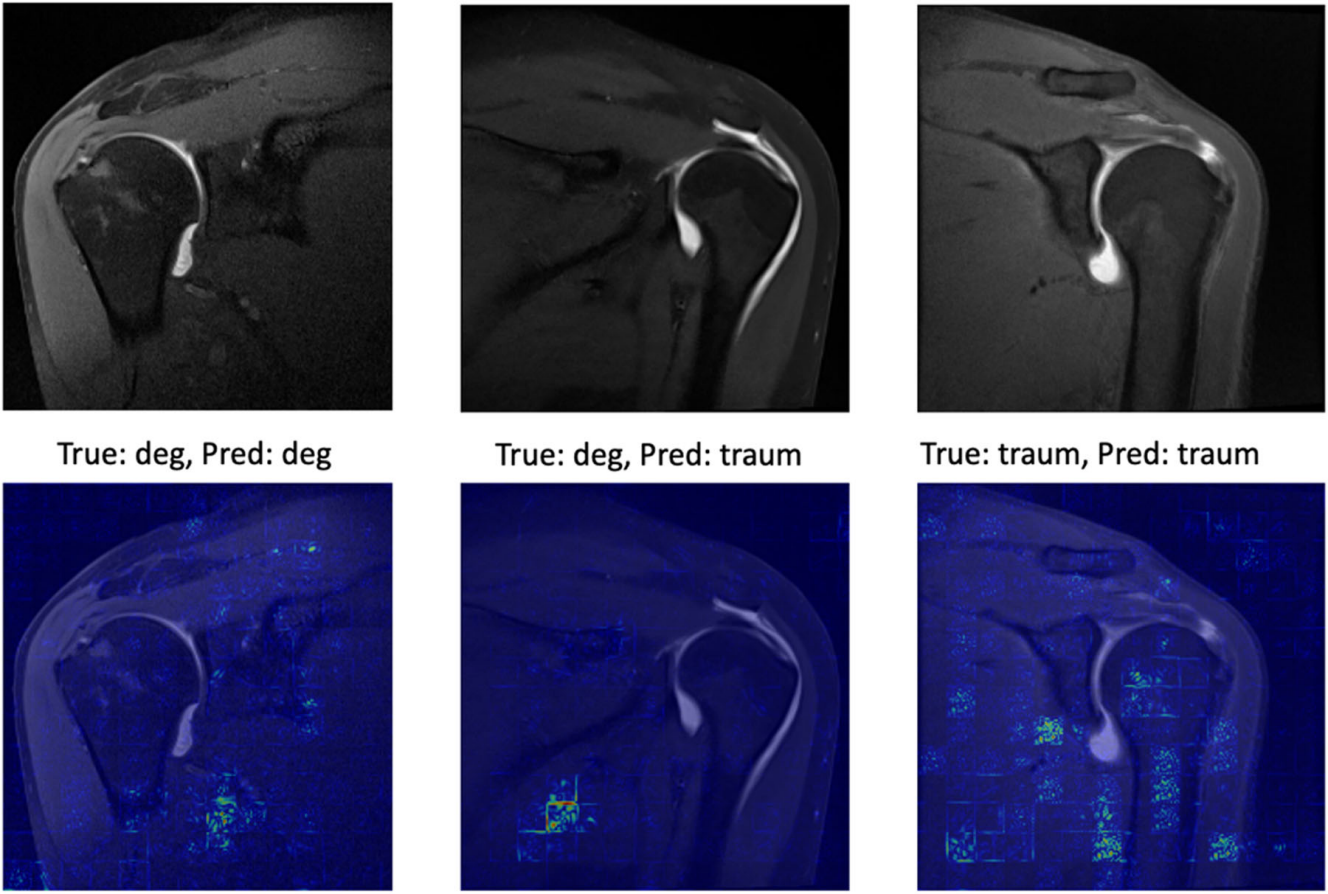
Saliency maps of correctly and incorrectly classified magnetic resonance imagings. There were no representative images of traumatic rotator cuff tears classified as degenerative.

Saliency maps derived from the ViT model were analysed to interpret the regions of interest influencing predictions. In degenerative RCTs, the model tended to highlight well‐defined areas in the subscapularis region, whereas in traumatic cases, the attention appeared more diffuse (Figure 3).

## DISCUSSION

The primary finding of this study was the successful demonstration that this specific multimodal ViT model performed well on this initial dataset. With an accuracy of 75% overall and a recall of almost 80% this model outperforms traditional radiographic ‘signs’ [6, 15]. While it has to be noted that the performance cannot be considered great, one has to account for the limited dataset as well as the age‐matched cohorts, as higher age is a well‐known predictor of degenerative RCT [7]. This model proves, however, that even with limited and demanding data a capable model can be engineered to improve distinction of a notoriously challenging diagnosis.

It is also noticeable of how much of a performance uplift is provided by adding basic patient demographics like age and BMI on top of the ViT transforming into a multimodal model. This most basic information led to an improvement of performance between −7% and 9% across various metrics. Considering the availability of these metrics and technological advancements, it would be unwise to dismiss this as irrelevant.

The saliency maps generated based on the ViT model revealed an interesting and somewhat surprising pattern. Anticipating that the model would primarily focus on the rotator cuff region, the attention patterns were more diffuse and variable, particularly in traumatic cases (Figure 3). This finding suggests that the model may be identifying subtle changes in the noninjured regions of the rotator cuff, beyond the traditionally examined cuff area, potentially incorporating broader anatomical context that human observers might overlook. For instance, the diffuse activation could reflect the model's sensitivity to widespread bone marrow oedema or global soft‐tissue inflammatory changes that are more indicative of an acute traumatic event. This is a departure from the human‐led search for specific markers like the ‘cobra sign’. The lack of consistent focus on the supraspinatus region itself raises questions about whether the current clinical focus on specific anatomical markers might be too narrow and suggests that future radiological research could benefit from a more holistic assessment of MRI scans in these cases.

The challenge of differentiating between traumatic and degenerative RCT has long been a significant clinical issue, as evidenced by previous literature [6, 14, 16, 25]. The recently introduced cobra sign, while valuable, represents just one of several radiographic indicators that clinicians have developed to aid in this distinction [6]. The current findings build upon this existing framework by demonstrating that AI can achieve superior accuracy compared to isolated radiographic signs, even in a stringently controlled, age‐matched cohort where differentiation is particularly challenging.

The presence of intramuscular oedema has been previously established as a highly specific marker for traumatic tears, achieving 100% specificity in one study [6, 15, 16]. This might contribute to the more diffuse activation map seen in traumatic RCT (Figure 3). However, the model's performance suggests that there may be additional, more subtle imaging features that contribute to accurate classification. This aligns with previous observations about intratendinous oedema being a sensitive, though not specific, indicator of traumatic tears and supports the notion that multiple imaging features must be considered in combination rather than in isolation [14, 16, 25]. Previous research has highlighted the limitations of relying on individual radiographic signs, such as the kinking sign or the presence of a residual tendon stump [6, 16]. The current study's results complement these findings by demonstrating that a ML approach can integrate multiple features simultaneously, potentially capturing complex patterns that might not be apparent through traditional radiological assessment. This is particularly relevant given the known difficulties in establishing clear morphological differences between traumatic and degenerative tears based on single imaging characteristics [6, 14, 16, 25].

### Limitations

This study has several important limitations that warrant careful consideration. First, the relatively small sample size of 99 cases, while sufficient for initial model development, limits the generalisability of the findings and cannot capture the full spectrum of even supraspinatus tear presentations. The single‐centre nature of this study further restricts the model's exposure to diverse patient populations. Second, despite stringent inclusion criteria, the retrospective design introduces inherent selection and recall biases, particularly regarding the accuracy of trauma history reporting and the timing of symptom onset. Third, the age‐matching strategy, while controlling for this crucial variable, possibly created an artificially balanced dataset that probably does not reflect the true clinical distribution of traumatic versus degenerative tears. The technical limitations of the model include the necessary standardisation of MRI slices to 20 centre slices, which might have resulted in the loss of potentially relevant information from excluded slices. Additionally, the reduction of image resolution to 224 × 224 pixels for model processing, though a standard practice for computational feasibility, could have obscured subtle imaging features in order to save computing power. From a methodological perspective, the binary classification approach may oversimplify the reality of rotator cuff pathology, where mixed or unclear etiologies are common. The exclusion of patients over 66 years and those with Goutallier Grade >II muscle atrophy, while methodologically sound, explicitly limits the model's applicability to these important patient populations, in whom the diagnostic question is often most pertinent. Furthermore, the focus on full‐thickness tears leaves questions about the model's performance in partial‐thickness tears unanswered. The lack of external validation on an independent dataset from different institutions and geographic regions remains a crucial limitation. The model's interpretability, while enhanced by saliency maps, still presents challenges in understanding the exact features driving its decisions, particularly given the unexpected attention patterns observed. Lastly, the study did not account for variations in MRI protocols, field strengths, or the potential impact of contrast administration timing in MR arthrography, all of which could affect image characteristics and, consequently, model performance.

While promising, the translation of this model into a real‐world clinical tool requires addressing several practical considerations. For widespread adoption, especially in nonacademic settings, the model must be accessible and easy to use. This could be achieved through integration into existing picture archiving and communication systems or via secure, cloud‐based platforms. Such an implementation would require significant investment in data infrastructure and would need to navigate regulatory pathways for approval as a medical device (e.g., from the FDA or via CE marking). Furthermore, future studies will need to demonstrate not only clinical utility but also cost‐effectiveness to justify its integration into standard diagnostic workflows. Although the technology is still in a developmental phase, these steps are crucial for its eventual journey from a research concept to a routine clinical support tool.

## CONCLUSION

This multimodal ViT model, achieving 75% accuracy in differentiating traumatic from degenerative RCTs, demonstrates the significant potential of AI to assist in a diagnostically challenging clinical scenario. The model's ability to integrate imaging and demographic data provides a promising foundation for a more robust CDS tool. While this pilot study is constrained by notable limitations, including its small sample size and single‐centre design, these shortcomings clearly define the path forward. The next critical steps involve validation on larger, multicentre datasets to ensure generalisability and further investigation into the novel imaging features highlighted by the model's saliency maps. Ultimately, translating this technology into clinical practice will require rigorous testing and seamless integration into clinical workflows, with the goal of providing a reliable tool that can support clinicians and improve patient outcomes.

## AUTHOR CONTRIBUTIONS

All listed authors have contributed substantially to this work. Felix C. Oettl and Pascal R. Furrer performed data extraction and literature review. Ali B. Malayeri performed engineering of the machine learning model. Primary manuscript preparation was performed by Felix C. Oettl. Editing and final manuscript preparation were performed by Ali B. Malayeri, Pascal R. Furrer, Karl Wieser, Philipp Fürnstahl and Samy Bouaicha. All authors read and approved the final manuscript.

## CONFLICT OF INTEREST STATEMENT

Karl Wieser has received consulting fees from Arthrex and Zurimed. The remaining authors declare no conflicts of interest.

## ETHICS STATEMENT

All procedures performed in studies involving human participants were in accordance with the ethical standards of the institutional and national research committee and with the 1964 Helsinki declaration and its later amendments or comparable ethical standards. Informed consent was obtained from all individual participants included in the study. Ethical approval for this study was obtained from the local ethical committee (Cantonal Ethics Committee Zürich, BASEC No. 2020‐02647).

## Data Availability

The data that support the findings will be available following an embargo from the date of publication to allow for commercialisation of research findings.
